# Biomarkers: A Definite Plus in Pneumonia

**DOI:** 10.1155/2009/675753

**Published:** 2009-11-16

**Authors:** Hanssa Summah, Jie-Ming Qu

**Affiliations:** Department of Pulmonary Medicine, Huadong Hospital, Fudan University School of Medicine, Shanghai 200040, China

## Abstract

During the past few years, biomarkers have emerged as an indispensible tool in the diagnosis of pneumonia. To find an ideal diagnostic biomarker for pneumonia is not an easy task. Not only should it allow an early diagnosis of the condition, but it should also allow differential diagnosis from other noninfectious conditions. Ongoing research is being done in this field so as to put an array of biomarkers at the disposal of doctors to improve the diagnosis of pneumonia when patients present to them with cough or nonspecific symptoms which could easily be misinterpreted as symptoms of other conditions. Procalcitonin and soluble triggering receptor expressed on myeloid cells-1 have emerged as reliable diagnostic markers in pneumonia, and are better when compared to other markers, namely, C-reactive protein, leukocyte count, and proinflammatory cytokines. Many other biomarkers are being studied for their probable use in diagnosing pneumonia but have yet to prove their benefit.

## 1. Introduction

Pneumonia has a high morbidity and mortality rate all around the world today [1]. Very often, clinical signs of pneumonia can be very elusive. At the heart of the dilemma, the question remains: “what is the fastest way to come to the correct diagnosis?” Because the faster the diagnosis is reached, the earlier the treatment begins. However, there is almost always a large lapse of time between the time of onset of symptoms and the start antibiotic therapy due to delayed diagnosis. In an attempt to achieve the rapid diagnosis of pneumonia and shortened antibiotic courses, an innovative approach is now being contemplated—the use of biomarkers. Till now, there is no universal definition of a biomarker, but it can be understood to be any biomolecule that is associated with a particular pathological or physiological state. Ideally, a biomarker should be one which cannot be detected or whose value is very low in the absence of inflammation; it should rise with increasing inflammatory processes and should decrease with resolving inflammation. 

 Physicians are becoming more and more interested in the use of biomarkers since there is no “gold standard” which is both sensitive and specific enough to help them reach the “correct” diagnosis. A “correct” diagnosis would be one in which the causative pathogen can be identified morphologically. However, 70% of patients with radiologically confirmed community-acquired pneumonia (CAP) do not have the causative organism identified. But to what extent should we rely on biomarkers to reach our diagnosis? In their recently published review article, P. Schuetz et al. stated that “only randomized controlled trials (RCTs), in which antimicrobial therapy is guided by specific cut off ranges of the biomarkers and in which the primary measure of efficacy is medical outcome, have the potential to evaluate the ultimate clinical usefulness of a diagnostic biomarker” [2]. Hence, the growing need of RCTs on biomarkers to evaluate their use in the diagnosis of pneumonia.

 Some of the biomarkers which are at the offing as an adjunct in the diagnosis of pneumonia include C-reactive protein, leukocyte count, immunoglobulins, and proinflammatory cytokines. There are other biomarkers whose importance is growing in the medical field. They are procalcitonin (PCT) and Triggering receptor expressed on myeloid cells-1 (TREM-1). This paper mainly focuses on C-reactive protein, procalcitonin, and Soluble Triggering receptor expressed on myeloid cells-1 (TREM-1). There are some other biomarkers which are still being studied for their probable use in pneumonia; these include copeptin, cortisol, endotoxin, proadrenomedullin, amongst others.

## 2. C-Reactive Protein (CRP)

C-reactive protein (CRP), identified in 1930, is an acute-phase protein. Whenever there is an infection or tissue inflammation, interleukin-6, interleukin-1*β*, and tumor necrosis factor-*α* stimulate hepatocytes to synthesise CRP. Within 4–6 hours of stimulation, CRP is secreted. Thereafter, its level doubles every 8 hours and reaches its maximum value at 36–50 hours. Once the stimulus is no longer present, the CRP value starts falling with a half-life of 19 hours [3]. For many years, the value of CRP in healthy individuals has been considered to be less than 0.5 *μ*g/mL [4]. However, in a large study carried out in two different countries (comprising of 2291 males and 2203 females), Hutchinson et al. found that CRP increases as the age of individuals increases [5]. Unfortunately, there has not been any further study to support these data. Ever since its identification, CRP has found a major place in screening for the presence of inflammation as well as in following the progression of disease activity. This is mainly due to the fact that CRP can be increased in a number of inflammatory processes, for example, pneumonia, pancreatitis, pelvic inflammatory disease, and urinary tract infections. Its level is also increased in meningitis, neonatal sepsis, and occult bacteremia [6]. 

 CRP, even though being nonspecific, has proved to be helpful in establishing the etiology of some infections. A high CRP value (>100 mg/L) can indicate a severe bacterial infection [7, 8]. Patients diagnosed with CAP were enrolled in a study so as to determine the role of CRP in differentiating CAP of different etiologies and in treatment guidance. The pathogens cultured from bronchoalveolar lavage (BAL) of these patients were *Streptococcus pneumonia* (*S. pneumonia*), viruses, *Chlamydia pneumoniae*, *Mycoplasma pneumoniae*, *Legionella pneumophila* (*L. pneumophila*), and *Coxiella burnetii*. Serum CRP values from these patients revealed to be valuable in the following ways: (1) CRP values were especially high in patients with pneumonias caused by *S. pneumoniae* or *L. pneumophila*; (2) CRP values increased with the severity of disease, showing that CRP can be used to predict the severity of disease; (3) CRP allowed the commencement of appropriate treatment [9]. Vázquez et al. showed that CRP values are higher in L. pneumophila infection than in CAP of other etiologies [10]. In 106 military conscripts who had pneumonia, CRP, with a cut-off value of 85 mg/L, could differentiate pneumococcal pneumonia from viral and mycoplasmal pneumonia. However, it was not useful in distinguishing viral from mycoplasmal infection [11]. High CRP levels have also been noticed in patients with bacteremia following pneumococcal pneumonia [12]. In subjects with hematologic malignancies, Offidani et al. found that the level of CRP is higher in those with fungal pneumonia than those with nonfungal pneumonia [13]. In drug-induced aspiration pneumonia, early measurement of CRP enables the diagnosis of aerobic bacterial content [14]. According to Smith and Lipworth, CRP can be used to differentiate between pneumonia and acute bronchitis [15]. 

 In children, it is even more important to establish the etiology of pneumonia as quickly as possible as any delay in treatment can have fatal consequences. Marcus et al. showed that in the pediatric emergency department the quick-read c-reactive protein test can be used to differentiate bacterial pneumonia from viral pneumonia [16]. In a meta-analysis of 1230 children, it was concluded that serum CRP concentrations above 40–60 mg/L are, albeit weak, predictors of bacterial etiology [17]. Heiskanen-Kosma and Korppi's, on the other hand, had a quite divergent opinion. According to them, CRP level is not associated with any microbial etiology of pneumonia in pediatric patients. In the study, they carried out in the Department of Pediatrics in Kuopio University Hospital in Finland, they enrolled 193 patients with pneumococcal infection, mycoplasmal and/or chlamydial infection and viral infections. The mean CRP concentrations (95% confidence interval) in these groups were 26.8 mg/L (20.1–33.5 mg/L), 31.8 mg/L (20.5–33.1 mg/L), and 26.1 mg/L (19.1–33.1 mg/L), respectively, and 24.9 mg/L (18.8–31.0 mg/L) in patients with no etiological findings [18]. A study conducted by Toikka et al. supported these findings [19]. When it comes to Ventilator associated pneumonia (VAP), Póvoa et al*. *showed that CRP (>9.6 mg/dL) has a good accuracy, with sensitivity of 87% and a specificity of 88% to diagnose VAP in a population of ICU patients. Further, high CRP levels are associated with poor outcome [20].

 Unfortunately, CRP does not allow the possibility to distinguish between all the types of pneumonia. In a systematic review published in BMJ in 2005, it was stated that “testing for C reactive protein is neither sufficiently sensitive to rule out nor sufficiently specific to rule in an infiltrate on chest radiograph and bacterial etiology of lower respiratory tract infection” [21]. However, the advantage of CRP is that CRP levels can be used for the follow-up of pneumonia as well as to evaluate patient management and response to antibiotic therapy [22]. 

## 3. Procalcitonin (PCT)

Procalcitonin (PCT), a 116-amino acid peptide, is produced by the c-cells in the thyroid and its concentration in the serum of healthy individuals is very low (<0.1 ng/mL). During microbial infection, there is an increase of CALC-I gene expression which causes a release of PCT from all parenchymal tissues and differentiated cell types throughout the body, including the liver and peripheral blood mononuclear cells [23]. The inflammatory release of PCT can be induced in two main ways: one is due to toxins released by microbes (endotoxin); and another one is through cell-mediated host response mediated by proinflammatory cytokines (e.g., interleukins 1b and 6, tumor necrosis factor-alpha). It should be noted that procalcitonin is also elevated in noninfectious conditions such as trauma, surgery, cardiogenic shock, burns, heat stroke, acute respiratory distress syndrome, infected necrosis after acute pancreatitis, and rejection after transplantation [24–30]. Today, PCT is known as a SMART biomarker for sepsis and infection since it satisfies all the required criteria, that is, (1) it has a high *specificity* and *sensitivity*, (2) it is readily *measurable*, (3) it is *affordable* and *available* in many hospitals, (4) it is *responsive* and *reproducible *(5) having half-life of 24 hours, it can be measured in a *timely* fashion [31]. 

 The widespread use of antibiotics for nonbacterial infections has led to antibiotic resistance. In order to reduce this phenomenon, antibiotic use must be limited to infections of bacterial etiology [32]. This is where PCT has proved its utility. Studies using the highly sensitive Kryptor assay have shown that PCT guidance can lead to the safe withholding of antibiotics among patients with low PCT levels (<0.25 *μ*g/L) and no clinical signs of severe illness. On the other hand, a PCT ≥0.25 and <0.5 ng/mL indicates possible bacterial infection and it is advised to initiate antibiotic therapy in these cases. A PCT ≥0.5 ng/mL is suggestive of the presence of bacterial infection and here antibiotic treatment strongly recommended. Using the values of PCT to predict the use of antibiotics does not only reduce the use of any unnecessary antibiotic usage but it also decreases the duration of therapy [31]. In 2004, Christ-Crain et al. showed that the use of PCT reduced antibiotic overuse in patients with lower respiratory tract infections. In their study, the risk of antibiotic exposure was reduced by 50% [33]. In a randomized trial to determine the length of antibiotic therapy using a laboratory parameter was carried out in 2006, Christ-Crain et al. successfully showed that using PCT as therapy guidance can actually reduce the length of therapy from 12 days to 5 days and the duration of antibiotic therapy was shortened by 65% with a similar outcome in patients independent of the severity of CAP [34]. A randomized, open, multicenter, noninferiority trial carried out from December 2004 to April 2006 showed that PCT can also be used to decrease antibiotic therapy outside the hospital setting [35]. 

 It has been found that the use PCT varies according to the severity of pneumonia. In patients with a low Pneumonia Severity Index (PSI, classes I-II), PCT can predict microbial etiology of pneumonia. In these patients, PCT level is higher in those with pneumonia of bacterial etiology than in those with pneumonia of nonbacterial etiology and hence appropriate treatment may be selected based on measurements of PCT. On the other hand, in patients with high PSI risk classes (classes III-V), PCT has proved to be a good prognostic marker rather than a diagnostic marker [36, 37]. It is well known that the diagnosis of pneumonia in children can be very difficult since the latter have nonspecific symptoms. Moreover, it is even more important to come to a quick diagnosis in children since delay in treatment commencement may have fatal outcomes in these patients. A study conducted in France in 1999 had the aim of comparing the use of PCT in children with the use of Interleukin-6, CRP, and Interferon-alpha for differentiating bacterial and viral infections. The results showed that in the pediatric emergency room, PCT is a reliable marker in the diagnosis of bacterial CAP [38]. Two years later, a study comprising of 101 children was carried out in Italy and it was found that a PCT level greater than 1.0 ng/L can help in distinguishing bacterial from viral pneumonia in children who are above 5 years of age [39]. A study conducted by Franzin and Cabodi showed that PCT is increased in Legionella pneumonia and, despite its nonspecificty, it can be used as a prognostic marker [40]. These results are in concordance with that from a study carried out by Haeuptle et al. [41]. 

 Patients on mechanical ventilation comprise another group of people in whom the diagnosis of pneumonia is very challenging. In these patients, clinical findings, saliva and tracheal specimen cultures are nonspecific when it comes to diagnosing pneumonia. Blood cultures and pleural fluid cultures have failed to show high sensitivities. PCT, however, has been a breakthrough in diagnosing pneumonia in this category of patients due to its high specificity for bacterial inflammation. Therefore, it is now considered a very useful adjunct in the diagnosis of VAP of bacterial origin [42]. During the past few years, there has been growing interest about how to predict patients who are at risk of having VAP so as to start early antibiotic therapy. Studies have been carried out to investigate whether PCT can be useful in this respect. Pelosi et al. have found that in patients requiring mechanical ventilation as a result of severe brain injury, measurements of serum PCT level when the patients are first placed in ICU, along with clinical pulmonary score infection score can be useful in predicting which patients will subsequently have VAP [43]. Ramirez et al. came up with the same findings and further added that CPIS and serum PCT below the cut-off point of 2.99 ng/mL are useful in excluding false-positive diagnosis of VAP since when used together they have a sensitivity of 100% [44]. Alveolar PCT, on the contrary, does not help in the early diagnosis of VAP [45]. There have been some studies which have shown that PCT is, in fact, not a good biomarker: Linssen et al. have found that PCT concentrations, in serum as well as in bronchoalveolar lavage fluids have no value when diagnosing VAP [46]. The poor diagnostic capacity of PCT in VAP has been further supported by Luyt et al. and in the same study, they concluded that levels of PCT must not be used to guide antibiotic therapy in patients with VAP [47]. 

 Immunocompromised patients are susceptible to various pulmonary infections and very often the signs and symptoms are nonspecific. They need a quick and accurate diagnosis to be able to start the appropriate treatment as soon as possible and thus increase the possibility of a favorable outcome. Stolz et al. studied BAL fluid neutrophils, serum PCT, and CRP to determine their use in the diagnosis of bacterial pulmonary infection in immunocompromised patients. In their study they confirmed that clinical signs and symptoms are not useful in the differential diagnosis of pulmonary complications in these patients. However, PCT, with a cut off value of 0.5 ng/mL had a specificity of 84%; CRP, with a cut off value of 20 mg/L had a sensitivity of 84% and a specificity of 48% [48]. It is very difficult to differentiate between tuberculosis and CAP in HIV-positive patients due to similar clinical presentations and radiographic changes. Schleicher et al. carried out a study to determine whether PCT and CRP levels in HIV-positive subjects can be used to differentiate between tuberculosis and pneumonia. They found that procalcitonin (level >3 ng/mL) and CRP (level >246 mg/L) help in predicting pneumococcal CAP in HIV-positive subjects [49]. 

 Ever since PCT has started gaining widespread use, clinicians have been questioning themselves about whether the use of PCT has made the use of CRP obsolete. Many studies have been conducted in order to compare the use of PCT with that of CRP in clinical practice. PCT can be used in the differential diagnosis of CAP from other conditions when there is an infiltrate on the radiograph. Müller et al. demonstrated that PCT and hsCRP improve the diagnosis CAP when used together [50]. In critically ill children, PCT has been found to be a better marker of infection as compared to CRP and leucocyte count. Moreover, a level of PCT greater than 2 ng/mL might be of use when differentiating severe bacterial infections from non-bacterial infections in children [51]. In a meta-analysis of 12 studies carried out by Simon et al., it was found that even in adults, PCT level is more sensitive (88% [95% confidence interval {CI}, 80%–93%] versus 75% [95% CI, 62%–84%]) and more specific (81% [95% CI, 67%–90%] versus 67% [95% CI, 56%–77%]) than CRP level for differentiating bacterial from noninfective causes of inflammation [52]. To further prove the superiority of PCT over CRP, Hedlund and Hansson carried out a study in patients being treated for CAP. They found that, with a cut-off point of 0.5 ng/mL, PCT but not CRP is able to help in the differentiation of typical bacterial pneumonia from atypical pneumonia [53]. 

 According to Holm et al., there is no indication that PCT is superior to CRP in indentifying patients with pneumonia, but they did mention that PCT may be superior to CRP when distinguishing mycoplasma and other bacterial infections [54]. PCT has been found to have a sensitivity of 100% and specificity of 75% in indicating VAP in patients seven days following successful cardiopulmonary resuscitation (cut off value of 2 ng/mL). The level of PCT was found to be elevated a median of 2 days before VAP was diagnosed clinically. Conversely, CRP has not been found to be valuable in this respect [55]. 

## 4. Soluble Triggering Receptor Expressed on Myeloid Cells-1 (sTREM-1)

Triggering receptor expressed on myeloid cells-1 (TREM-1) is another biomarker which holds great promise when it comes to pneumonia. TREM-1 belongs to the immunoglobin superfamily and is involved in inflammatory response. It is expressed on the surface of neutrophils, monocytes, and macrophages during acute inflammatory responses. It has been found that the tansmembrane adapter protein DAP12 is the signal transduction molecule through which TREM-1 activates neutrophils and monocytes [56]. It has the advantage of being increased during infectious processes but not in noninfectious inflammatory conditions like psoriasis, ulcerative colitis, and vasculitis. TREM-1 exists in both a membranous and a soluble form (soluble triggering receptor expressed on myeloid cells-1; sTREM-1) [57]. 

 Levels of sTREM-1 have been found to be elevated in bronchoalveolar lavage fluids of patients with pneumonia, in the plasma of septic patients, and in the exhaled breath condensate in VAP patients [58]. However, no significant difference has been found in the levels obtained from CAP and VAP patients. In a review published in Clinical Medicine and Research, Gibot reported sTREM-1 levels ≥5 pg/mL were measured in BAL fluid from approximately 95% of CAP patients and 100% of VAP patients as compared to 10% of patients without pneumonia. The area under the ROC curve to determine whether or not the patients had pneumonia was 0.93. A cut-off value of 5 pg/mL had a sensitivity of 98% and a specificity of 90% to predict pneumonia [59]. Tejera et al. found that serum sTREM-1 is high in patients with CAP, and that the prognostic value of sTREM-1 is independent of age, other inflammatory markers such as IL-6, Pneumonia Severity Index, CURB-65, severity of sepsis, and nutritional status. They also found that patients who had increased sTREM-1 on admission had the worst prognosis [60].

In the absence of a good biomarker, all patients with pulmonary aspiration syndrome receive antimicrobial therapy when antimicrobial therapy should, in fact, be given only to those who have infectious pneumonitis. The use of sTREM-1 to differentiate between aspiration pneumonia and aspiration pneumonitis has been investigated by El Solh et al. However, BAL fluids in pulmonary aspiration syndrome subjects with a positive culture have been found to have a higher content of sTREM-1 (344.41 ± 152.82 pg/mL) than those who had a negative culture (142.76 ± 89.88 pg/mL; *P* < .001). The cut-off value of alveolar sTREM-1 was 250 pg/mL. Alveolar sTREM-1 was found to have a sensitivity of 65.8% (95% CI 48.6–80.4) and a specificity of 91.9% (95% CI 78.1–98.2) in distinguishing between the two conditions [61]. In a study carried out by Richeldi et al., BAL specimens of patients with CAP, tuberculosis, and interstitial lung diseases were collected. It was found that TREM-1 expression is significantly increased in lung neutrophils and lung macrophages of patients with pneumonia caused by extracellular bacteria as compared to patients with pulmonary tuberculosis or interstitial lung diseases [62]. The presence of sTREM-1 in BAL fluid may also be useful in diagnosing bacterial or fungal pneumonia. In a study of 148 patients who were being mechanically ventilated, it was found that sTREM-1 had a sensitivity of 98% and specificity of 90% for the diagnosis of bacterial and fungal pneumonia [63, 64]. This was in concordance with the study carried out in 28 critically ill mechanically ventilated patients where Determann et al*. *found that sTREM-1 has a sensitivity of 75% and specificity of 84% in diagnosing pneumonia. In patients who developed VAP, sTREM-1 levels in BAL were found to start rising 6 days prior to VAP diagnosis. The greatest increase was 2 days prior to diagnosis. As for non-VAP patients, sTREM-1 levels showed no significant change during the study period [65].

 The use of exhaled ventilator condensate (EVC) to diagnose VAP is being contemplated more and more nowadays. EVC has the advantage of being noninvasive as compared to BAL. EVC is easily collected in the expiratory line of patients receiving mechanical ventilation. This technique was used by Horonenko et al. and they successfully demonstrated that when clinically in doubt of VAP, sTREM-1 in EVC may be of use in establishing the diagnosis of pneumonia [66]. It is important to detect CAP patients who are unresponsive to treatment at an early stage so as to be able to change the treatment regimen. In a study conducted in Taiwan, Chao et al. used serial changes of CRP and sTREM-1 to show that sTREM-1 can be used in addition to CRP to detect nonresponsive patients [67]. sTREM-1 cannot be used on its own to diagnose pneumonia, but it can be used as a complementary tool to reinforce the usual diagnostic work-up. Early treatment can be started in VAP patients when sTREM-1 is used. Furthermore, Gibot et al. stated that combined measurement of serum PCT and BAL sTREM-1 can be useful in differentiating VAP from extrapulmonary infection [68].

## 5. Other Biomarkers

A variety of other biomarkers, namely, endotoxin, proadrenomedullin, natriuretic peptides, endothelin-1 precursor peptides, as well as copeptin and cortisol levels, are being studied so as to improve the diagnosis and prognosis of pneumonia [69]. Here, it is important to point out that biological markers are only to be used as a complementary tool to reach diagnosis and they, in no way, replace the clinical importance of diagnosis. The biomarkers are useful in guiding culture sampling, empirical antibiotics prescription, following clinical course of the condition and identify those who do not respond to therapy [70]. We would also like to point out that while Endotoxin is a diagnostic marker just like CRP, PCT, and sTREM-1, the other biomarkers mentioned (i.e., proadrenomedullin, natriuretic peptides, endothelin-1 precursor peptides, as well as copeptin and cortisol levels) have up to now only been found to be useful as prognostic markers and may be of great help in the risk stratification of patients.

## 6. Endotoxin

Endotoxin measurements in BAL showed a relation between its concentration and the quantity of Gram-negative bacteria in BAL fluids of VAP patients. Endotoxin allows rapid diagnosis of Gram-negative bacterial pneumonia [71]. Flanagan et al. found that endotoxin level within four days of starting mechanical ventilation is an accurate as well as a rapid way for diagnosing VAP [72]. In a study conducted by Nys et al., it was established that antimicrobial Gram-negative therapy may be justified according to the results of endotoxin level in BAL fluids of ventilated patients having pneumonia [73].

## 7. Copeptin and Midregional Pro-Atrial Natriuretic Peptide (MR-proANP)

First described in 1972 by Holwerda, copeptin is a 39-amino acid glycosylated peptide with a leucine-rich core segment. Copeptin, along with antidiuretic hormone arginine vasopressin (AVP) derive from a precursor protein, a 164-amino acid known as pre-pro-vasopressin, which consists of a signal peptide, AVP, neurophysin II, and copeptin [74–77]. Copeptin is the C-terminal part of pro-AVP. Copeptin remains stable in withdrawn blood for days and its level can be measured quickly and easily [78]. Copeptin levels in blood have already been shown to be of use in the diagnosis of diabetes insipidus and in the monitoring of sepsis and cardiovascular diseases [79]. Copeptin has the advantage of not varying with age. In a study conducted on 359 healthy individuals, copeptin levels ranged from 1 to 13.8 pmol/L, with a median of 4.2 pmol/L, and were detectable in 97.5% of the individuals. However, it has been found that its level is significantly different between the two sexes, being higher in men than in women. In the very same study, levels of copeptin were found to vary with exercise, fasting, and water load [78].

 Müller et al. have found that copeptin levels have a tendency to increase as the severity of lower respiratory tract infection increases and this increase has been found to be more pronounced in patients with CAP. Hence, copeptin may be useful in the risk stratification of patients with lower respiratory tract infections [80]. When investigating the correlation of copeptin with the severity of septic status in patients with VAP, Seligman et al. also found that copeptin increases progressively with severity of sepsis [81].

Atrial natriuretic peptide, primarily produced in the cardiac atria, belongs to the natriuretic peptide family [82]. The mid-region of the prohormone of ANP, known as MR-proANP (midregional pro-atrial natriuretic peptide) has been found to increase with severity of sepsis and may be used as a predictor of mortality in VAP [83]. It has been found that MR-proANP can be used as a prognostic marker in pneumonia [84].

The Bach study is a worldwide multicentre study with over 1600 patients. It shows that the use of PCT together with Mid-regional Pro-atrial Natriuretic Peptide (MR-proANP) supports the differential diagnosis of pneumonia and congestive heart failure [85]. B-natriuretic peptides, a well-established biomarker belonging to the natriuretic peptide family, may be used in the emergency department to differentiate dyspnea of pulmonary origin from that of cardiac origin [86].

 Both MR-proANP and CT-proAVP (C-terminal pro-vasopressin- Copeptin) can be used to predict severity of disease in patients with CAP [87].

## 8. Cortisol

In early postoperative patients, posttrauma, or sepsis patients, serum cortisol level rises. This has the benefit of redistributing effectively energy to the vital organs [88]. High cortisol levels were already known to be a good prognostic marker in sepsis. However, whether or not cortisol levels can be used as a marker of prognosis in CAP was still unknown. In order to investigate this, Christ-Crain et al. conducted a study in 278 patients with CAP. They found that initial measurements of cortisol levels (both free and total cortisol) were good markers of severity and prognosis. They were in fact as good as PSI and even better than laboratory parameters [89]. 

 As for proadrenomedullin and endothelin-1 precursor peptides, they have been found to be of great use in the risk stratification of patients with CAP [90, 91].

## 9. Conclusion

The array of biomarkers available to diagnose pneumonia and to aid its differential diagnosis from other diseases has brought a new turn in medicine. At one time, doctors had to rely on clinical findings and radiological findings and thus, there was much delay in treatment initiation. Biomarkers have improved both diagnosis and prognosis. However, biomarkers do not make clinical findings, radiological findings, and appropriate cultures obsolete. In fact, biomarkers are to be used only as an adjunct to diagnosis [92].
